# Predictors of weight loss outcomes in obesity care: results of the national ACTION study

**DOI:** 10.1186/s12889-019-7669-1

**Published:** 2019-10-30

**Authors:** Nikhil V. Dhurandhar, Theodore Kyle, Boris Stevenin, Kenneth Tomaszewski, The ACTION Steering Group, Lee M. Kaplan, Angela Golden, Kimberly Jinnett, Ronette L. Kolotkin, Michelle Look, Joseph Nadglowski, Patrick M. O’Neil, Thomas Parry, Søren Kruse Lilleøre, Novo Nordisk

**Affiliations:** 1 Texas Tech University, Lubbock, TX USA; 2 ConscienHealth, Pittsburgh, PA USA; 3 Novo Nordisk Inc., Plainsboro, NJ USA; 4 KJT Group, Inc., Honeoye Falls, NY USA

**Keywords:** People with obesity, Obesity attitudes, obesity management, Weight-loss success

## Abstract

**Background:**

A key objective of this study was to examine obesity care attitudes and behaviors of people with obesity (PwO) and determine independent factors associated with a self-reported sustained weight loss success outcome.

**Methods:**

An online survey was conducted in 2015 among 3008 U.S. adult PwO (BMI > 30 through self-reported height and weight). Multivariate logistic models explained variation in weight loss success, defined as ≥ 10% weight loss in previous 3 years and maintained for > 1 year.

**Results:**

Controlling for weight changes over time, we found significant associations between self-reported weight history and weight loss success. PwO who had personal motivation to lose weight, were willing to talk to a diabetes educator about their weight, who had their weight loss attempts recognized by a healthcare provider, and were diagnosed with “obesity” or “overweight” were more likely to report having success losing weight.

**Conclusions:**

This study does not determine causality, but suggests motivation and engagement with PwO may impact weight loss, and presents a basis for assessing the mechanism involved. Determining such mechanisms may identify important targets to improve obesity treatment outcomes.

**Trial registration:**

This study is registered with ClinicalTrials.gov, number NCT03223493, https://clinicaltrials.gov/ct2/show/NCT03223493. Registered July 17, 2017 (retrospectively registered).

## Background

Obesity is a serious, complex, and chronic disease that requires lifelong management [1–3]. This multifactorial condition is widely prevalent; more than one-third of the United States adult population has obesity, as defined by a BMI ≥ 30 [4]. Achieving a sustained weight loss can be difficult for many people with obesity (PwO) [5–8]. Many modifiable and non-modifiable factors may contribute to successful weight loss, including genetics, weight loss approaches, psychosocial factors, motivation levels, and available support system [9–14]. It is important to focus on modifiable factors contributing to weight loss success.

To better understand factors associated with weight loss outcomes, we examined survey results in a nationally representative sample of persons with obesity (PwO) from the ACTION (Awareness, Care, and Treatment In Obesity maNagement) Study [15]. A key objective of ACTION was to determine independent factors associated with a self-reported sustained weight loss success outcome among PwO. This study provides insight into the demographic, behavioral, and attitudinal factors associated with weight loss among PwO and actions health care providers (HCPs) may take to help their patients with obesity increase their chances for weight loss success.

## Methods

### Study design

This cross-sectional study, sponsored by Novo Nordisk Inc. and approved by an Institutional Review Board [16], was conducted in 2015 (from October 29th to November 12th) among US adults age ≥ 18 with obesity (defined as self-reported body mass index (BMI) of 30 kg/m^2^ or higher). PwO responded to an online survey which assessed obesity-related attitudes and behaviors and was developed based on literature review and qualitative research [17]. Respondent-level weighting was applied [18] to ensure the sample was representative of the U.S. population [19]. Except for participant characteristics, the data presented in this paper are weighted unless otherwise specified. A detailed methodological description has been previously published [15]. Five-point end-anchored scales assessed agreement, where “1” meant “do not agree at all,” and “5” meant “completely agree.” Responses of “4” or “5” were coded and reported as “agree” unless otherwise noted.

### Data analysis

A self-reported weight loss success outcome was defined within the survey instrument explicitly. A multivariate logistic regression model assessed variation in “weight loss success” (dependent variable) defined as: 1) *Weight loss history*: at least 10% weight loss in the previous 3 years; and 2) *Success*: weight loss at the time of survey response that was maintained for at least 1 year (by respondent self-report).

Bivariate associations between the outcome of interest and 140 possible independent variables were assessed and grouped into 3 domains: demographic, attitudinal, and behavioral. Independent variables with large bivariate effect sizes and significant practical implications were identified. Variance inflation factors were used to assess the degree of multicollinearity present among the remaining 32 independent variables; no variables were removed at this step.

To achieve a parsimonious model, a purely statistical approach was used to reduce the inputs. A Bayesian variable selection approach was used to overcome the biases and shortcomings of stepwise variable selection. A logistic regression model was estimated using the nine remaining independent variables; two were removed for non-statistical significance and high degree of correlation with other independent variables (feelings after most recent discussion of weight with HCP: supported; barriers to initiating a weight loss effort: my lack of motivation, respectively). Seven characteristics taken from demographic, attitudinal and behavioral survey domains were included in the model. Six of the seven variables included in the final model were statistically significant at the 5% level of significance; discussing weight with a diabetes educator was not statistically significant but increased the stability of the model.

A sensitivity analysis was conducted to determine the independent impact each variable in the model has on the average chance of being successful in losing weight and keeping it off for 1 year. To conduct the sensitivity analysis, we implemented the following procedure for each independent variable in the model: 1) for categorical variables, a 1000-iteration bootstrap sample (a technique of randomly sampling the data and iteratively calculating the statistics to generate more accurate population estimates) was performed to calculate the mean predicted probability of sustained weight loss success for incremental improvements and deteriorations of 1 to 100%; 2) for continuous variables, the mean predicted probability of sustained weight loss success was calculated for incremental improvements and deteriorations of 1 to 100%.

## Results

Adult PwO (*n* = 3008) completed online surveys; characteristics for PwO are displayed in Table 1.

**Table 1:** Sample characteristics (unweighted)

	People with Obesity
	(n = 3008)
Characteristics	*n* (%)
Sex	
Male	1378 (46)
Female	1630 (54)
Age	
Mean Age in Years (SD)	54.4 (14.3)
BMI Classification	
Class I (BMI 30- < 35)	1304 (43)
Class II (BMI 35- < 40)	896 (30)
Class III (BMI ≥ 40)	808 (27)
BMI	
Mean (kg/m^2^) (SD)	37 (6)

*SD* Standard deviation, *BMI* Body mass index

Slightly less than one-quarter of PwO (23%) reported at least 10% weight loss from maximum weight in the past 3 years to their current weight. Among these PwO, 44% reported having maintained weight loss for at least 1 year, representing 10% of all PwO surveyed.

### Multivariate model results

Model results are described in Table 2. The odds of sustained weight loss success are compared to a base case PwO who weighed 248 pounds 1 year ago and 221 pounds 10 years ago, and who has been formally diagnosed with obesity, reports their HCP not often recognizing the PwO’s previous weight management efforts, does not agree that he/she is motivated to lose weight, does not agree that his/her lack of motivation is a barrier to initiating a weight loss effort, and has not discussed or would not want to discuss weight with a diabetes educator.

**Table 2:** Multivariate Logistic Model Results - Variables and Odds of Sustained Weight Loss Success Compared to Base Case PwO^a^

Variable Type	Variable Description	Variable Specification^b^	Coefficient (Standard Error)	Odds Ratio and 95% Confidence Interval	PwO Affected
Intercept	N/A	Constant	−3.67 (0.4847)	N/A	N/A
Demographic	Weight History 1 year ago	Continuous	−0.01 (0.0026)***	0.99 (0.99–1.00)^c^	Mean = 248lbs
Demographic	Weight History 10 years ago	Continuous	−0.02 (0.0021)***	1.02 (1.01–1.02)	Mean = 221lbs
Demographic	Formal diagnosis of obesity	No, Not sure [2, 3] - > 1 Yes [1] - > 0	−0.45 (0.1902)*	0.64 (0.44–0.92)	No/Not sure [2, 3] = 44%
Attitudinal	When discussing your weight with your HCP, how often do they recognize your previous weight management efforts?	Often [4, 5] - > 1 Not often [1–3] - > 0	0.69 (0.1687)***	1.99 (1.43–2.77)	Often [4, 5] = 39%
Attitudinal	Attitudes toward weight loss: I am motivated to lose weight	Agree [4, 5] - > 1 Do not agree [1–3] - > 0	0.59 (0.1746)***	1.81 (1.28–2.54)	Agree [4, 5] = 45%
Attitudinal	Barriers to initiating a weight loss effort: my lack of motivation	Agree [4, 5] - > 1 Do not agree [1–3] - > 0	−0.49 (0.1699)**	0.61 (0.44–0.86)	Agree [4, 5] = 52%
Behavioral	HCPs discussed weight with/would discuss weight with: diabetes educator	Yes [1] - > 1 No [2] - > 0	0.20 (0.2317)	1.22 (0.78–1.93)	Yes [1] = 10%
Model Fit Statistics					
	Mean (95% Confidence Interval)				
Accuracy	70% (65–74%)				
Specificity	70% (54–79%)				
Sensitivity	66% (52–75%)				

*PwO* People with Obesity, *HCP* Healthcare Provider, *Lbs* Pounds

^a^Base Case PwO: weight 1 year ago = 248 lbs., weight 10 years ago = 221 lbs.; formal diagnosis of obesity = yes, HCP recognizes previous weight management efforts = not often, I am motivated to lose weight = do not agree, my lack of motivation is a barrier to a weight loss effort = do not agree, discussed/would discuss weight with diabetes educator = no

^b^Answer categories and scale definitions

*p < 0.05, **p < 0.01, ***p < 0.001

^c^Due to rounding; unrounded value is < 1.00

The model’s statistical fit was assessed by using the model diagnostics of accuracy (percentage of the time the model accurately classifies a PwO as successful in achieving sustained weight loss), specificity (percentage of failed weight loss attempts accurately predicted by the model), and sensitivity (percentage of successful weight loss attempts accurately predicted by the model). Individual observations were assessed for their degree of leverage/influence and residuals were assessed via a studentized residuals vs. fitted probability plot. Higher leverage points were dispersed throughout the fitted probability space – these points were not overly influencing prediction to one class or the other. The points with the highest residual error were all cases of successful weight loss and occurred at the lower end of the fitted probability scale (i.e. 0.0–0.2) indicating that a low probability threshold should be used for classification of successful weight loss. A threshold of 0.11 was identified as the boundary that best-balanced model accuracy (70%), sensitivity (68%), and specificity (70%). Two-thousand (2000) splits of the data into 80% training and 20% test showed stable model performance at this threshold with values as shown in Table 2.

Self-reported weight 1- and 10- years prior were both significantly associated with sustained weight loss success, controlling for beginning and ending weight (1 and 10 years prior, respectively); i.e., the model controlled for variation associated with varying weights. On average, each additional pound of weight 1 year ago predicted a decrease in the success odds by ~ 1%; however, each additional pound of weight 10 years ago increased the odds of success by a factor of 1.02 (increasing the odds by ~ 2%).

Not formally being diagnosed with obesity was associated with a decrease in PwO’s odds of sustained weight loss success by a factor of 0.64. PwO who felt their lack of motivation was a barrier to weight loss had lower odds of reporting success, even after controlling for other factors such as their changes in weight and attitudes towards initiating weight loss.

Discussion with a diabetes educator (or willingness to discuss) was associated with increased odds of sustained weight loss success by a factor of 1.22. HCPs’ recognition of PwO’s previous weight loss efforts and PwO’s self-reported motivation to lose weight had the greatest impact on the odds of success, increasing them by a factor of 1.99 and 1.81, respectively.

To further illustrate how the odds of successful sustained weight loss may be manifested at the individual level, we explored several hypothetical scenarios (Table 3).

**Table 3:** Practical Scenarios and Impact on Baseline Prediction of the Logistic Model^a^

Practical Scenarios Defined			
Variable Description	Active HCP; Unmotivated PwO	Inactive HCP; Motivated PwO	Encouraging HCP; Motivated PwO
Formal diagnosis of obesity	Yes	No	No
HCP often recognizes PwO’s previous weight management efforts	Yes	No	Yes
PwO motivated to lose weight	No	Yes	Yes
PwO lack of motivation is a barrier	Yes	No	No
PwO discussed weight with/would discuss weight with diabetes educator	Yes	No	Yes
Practical Scenario Impact	Active HCP; Unmotivated PwO	Inactive HCP; Motivated PwO	Encouraging HCP; Motivated PwO
Predicted Probability of Sustained Weight Loss Success	12.20%	9.36%	22.84%
% Change from Population Average Prediction	6%	−19%	98%

*PwO* People with Obesity, *HCP* Healthcare Provider

^a^Population average prediction = 11.5%; weight history 1 year ago and 10 years ago were set to the population average of 248 pounds and 221 pounds, respectively

The presence of PwOs' motivation in the absence of a formal obesity diagnosis or recognition of previous weight management efforts (“Inactive HCP; Motivated PwO”) was associated with a predicted 19% decrease in the rate of sustained weight loss success compared with the population average. A PwO who was unmotivated to lose weight but receives a formal diagnosis, has a discussion with a diabetes educator, and receives recognition from an HCP (“Active HCP, Unmotivated PwO”) is predicted to have a greater rate of success, with a 6% increase over the population average prediction. Finally, PwO who receive recognition of previous weight loss efforts from HCPs and report feeling motivated (“Encouraging HCP; Motivated PwO”) are more likely to have success, with a 98% increased likelihood compared with the population average.

### Sensitivity analysis

In the sensitivity analysis described above, we found that increasing obesity diagnoses rates and discussions with a diabetes educator have the least independent influence on the predicted success rate across the sample. Increasing the rate of diagnosis or discussions with a diabetes educator by 50% results in an absolute change of < 1% point from the baseline prediction of sustained weight loss success of 11.5% (baseline prediction is the model’s estimated sustained weight loss success at the means for each independent variable). A decrease in the prevalence of PwO’s self-reported motivation or a reduction in the prevalence of motivation as a barrier by 50% results in an absolute change of 1.4% points and 1.1% points from the baseline prediction, respectively. Increasing the proportion of PwO whose previous weight management effort is recognized by HCPs by 50% improves the average chance of success by 1.8% points.

**Fig. 1:**
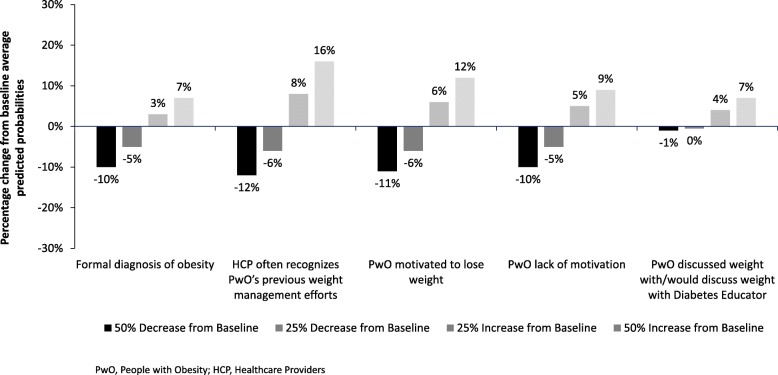
Percent Change from Baseline Average Success Rate. Increasing the rate by 50% relative to the average baseline rates yields improvement in the rate of success by as much as 16%

Although the absolute point changes in the average predictive probabilities are modest, these translate to substantial population level increases (number of PwO) relative to the average baseline rates of weight loss success (Fig. 1). Increasing the rate at which a PwO’s previous weight management efforts are recognized by an HCP by 25% is predicted to result in an 8% improvement in the rate of success. The same level of increase in a PwO’s self-reported motivation to lose weight is associated with a 6% improvement in the weight loss success rate.

### Relationship between BMI and attitudes towards weight loss discussions

Looking outside the model, we further sought to understand if weight loss discussions and attitudes towards them are associated with the BMI of PwO. Evaluating PwO by obesity class (Class I: BMI 30- < 35, Class II: BMI 35- < 40, Class III: BMI ≥ 40) [20], we found significant differences in attitudes and behaviors. PwO with Class III Obesity were significantly more likely (30%, 95 CI [26–35%]) than those with Class I (21%, 95 CI [18–24%], *p* < .001) or Class II Obesity (22%, 95 CI [18–25%], *p* < .001) to report having sought support from their HCP regarding weight management after they have tried losing weight on their own with no success. PwO with Class I or Class II Obesity (39.1%, 95 CI [35–43%] and 39%, 95 CI [35–43%], respectively) were significantly more likely than those with Class III Obesity (31%, 95 CI [27–35%]) to report not seeking support from their HCP (*p* = .002 and *p* = .003, respectively). Conversations with HCPs differed by obesity class; when asked who typically brings up weight during the appointment, PwO with Class I Obesity were significantly more likely to say they, rather than their HCP, typically started the conversation about their weight (51%, 95 CI [47–55%] than those with Class II (45%, 95 CI [41–50%, *p* = 0.026] and Class III Obesity (43%, 95 CI [39–47%], *p* = .002. Those with Class II and III Obesity were significantly more likely than those with Class I Obesity to report negative feelings of “embarrassed,” “discouraged,” and “blamed” after their most recent discussion with their HCP. When asked how their HCP could better support them in achieving a healthy weight, PwO with Class II and Class III Obesity were significantly more likely than those with Class I Obesity to cite “be more understanding of the challenges of losing weight,” “be more understanding of the challenges with living with obesity,” and “refer me to a dietitian.”

## Discussion

Although causation between the self-reported factors and weight loss outcomes cannot be implied from this observational study, the results from these analyses provide greater insight into factors that may predict a PwO’s chance of being successful in weight management, including the role of the HCP as one of these factors. The study offers an opportunity to understand the expectations and efforts of PwO around their struggle with the disease. The multivariate model revealed a sizable impact on sustained weight loss success on an individual level; the sensitivity analysis demonstrated a lower impact on a population level at the levels we tested. However, these small changes resulted in substantial relative impacts when considered at the population level.

Higher self-reported weight 1 year ago had a moderately negative impact on successful weight loss outcomes, indicating that PwO are more likely to achieve success over a longer period. This is supported by the positive impact of a higher weight on a longer time scale of 10 years. The greater the weight 1 year ago, the harder it is for PwO to be successful in their weight management efforts. More recent weight gain may be more of a challenge to PwO; struggling with obesity for a longer time may be a driving factor in PwO being more determined to make a serious weight loss attempt. Inversely, recent progress towards weight management goals (lower self-reported weight a year ago) may be a self-reinforcing mechanism: being closer to a goal in the near term makes reaching that goal more feasible. These variables can more generally be considered “control” variables, thus ensuring other model parameters have an independent effect after controlling for weight history.

Discussions with HCPs who acknowledge and support previous weight loss efforts were reportedly associated with substantially improved odds of an individual PwO’s successful weight loss attempt. Despite relatively low levels of improvement in the population, recognizing PwO’s weight loss attempts would require marginal efforts by HCPs and may have an exponential positive impact on the PwO-HCP relationship and the individual PwO’s chances for success. We hypothesize that the individual success rate improvements when HCPs engage in this behavior with their patients is driven by communicating support and encouragement for the patient.

Motivation is a key to PwO being successful in sustained weight loss [21–23]; at the start and during weight loss efforts, motivation remains independently relevant. An individual PwO’s lack of motivation, specifically with respect to *initiating* weight loss efforts, was associated with poorer outcomes. Additionally, PwO who were motivated during the weight loss process see a very large improvement in their odds of success. However, our model indicates that lack of motivation may be overcome or mitigated with proactive measures by an HCP including diagnosing patients with obesity, recognizing previous weight management efforts, and discussion with a diabetes educator, regardless of how successful the PwO may have been. This study suggests that even when a PwO is less motivated and perceives their lack of motivation is a barrier to sustained weight loss, engaged HCP interaction may result in “average” or even above average outcomes at the individual level; however, this relationship would need to be further explored in other research.

Formally diagnosing individual patients with obesity is also associated with weight loss success; this research may encourage HCPs to be engaged in supporting PwO in their weight loss efforts. As such, an appropriate diagnosis is a required first step for treating any disease. In addition, making a diagnosis may also be an effective avenue for HCPs to start the obesity management conversation with their patients with obesity, particularly those who have struggled with obesity for a long time.

Having discussed weight with, or being willing to discuss weight with a diabetes educator is also positively associated with successful weight loss, although the lack of independent statistical significance suggests this result is directional. The impact of discussing weight with a diabetes educator speaks to the benefit of additional support services and resources that can address co-morbid conditions associated with obesity.

Having regular discussions with PwO about their weight and weight management efforts is an important factor in effective obesity management [24, 25]. Understanding that PwO tend to seek care from their HCP only after their own self-management attempts have failed, as seen in this study, could help address and reduce stigma and bias in treating PwO. Recognizing previous weight management efforts could be an effective tool in ensuring weight management discussions are positive in nature and reduce negative feelings among PwO, especially those with Class II or Class III Obesity. By acknowledging the daily and often life-long struggles of PwO, HCPs may convey a greater sense of support that may help increase PwO’s motivation and chances of weight loss success. Lastly, treating obesity as a multi-factorial disease by ensuring PwO have access to a broader clinical team may support patients’ obesity management efforts.

Limitations of this study have been previously reported including the cross-sectional design and self-reported nature of height and weight being potential limiting factors [15]. An additional limitation was the use of a self-reported assessment of motivation rather than a validated instrument specifically designed to assess this factor. Selection of a logistic regression model assumes that the probability of sustained successful weight loss is roughly approximated by a logistic distribution in the population; however, logistic regression is the most common empirical model of binary dependent variables across disciplines [26].

## Conclusion

Weight loss success was consistent with PwO-reported weight loss history; predictions from our logistic regression model consistently replicate success rates based on PwO-reported weight loss history. Even after controlling for weight history, motivation in addition to support from HCPs is associated with weight loss success among PwO. This study highlights the role that personal motivation and engagement of PwO may play in weight loss success and sets the stage for further investigation of these factors in predicting success. Such an understanding may aid in the identification of key approaches to improve obesity management and outcomes.

## Data Availability

Data will be shared with bona fide researchers submitting a research proposal requesting access to data. Data will be shared for use as approved by the Independent Review Board according to the IRB Charter (see novonordisk-trials.com). Access request proposal form and the access criteria can be found at novonordisk-trials.com. Individual participant data will be shared in data sets in a de-identified/ -anonymized format. The data will be made available on a specialized SAS data platform.

## Abbreviations

ACTION Study: Awareness, Care, and Treatment In Obesity maNagement
BMI: Body Mass Index
CI: Confidence Interval
HCP: Healthcare Provider
Lbs: Pounds
PwO: People with Obesity
SD: Standard deviation
